# Astrocytic connexins in alcohol use disorder: mechanisms of neuroinflammation and therapeutic potential

**DOI:** 10.3389/fpsyt.2026.1799469

**Published:** 2026-06-03

**Authors:** Esohe Imafidon, Elise Allan-Le, Kafui Dzirasa, S. Alex Marshall, Julia Derk

**Affiliations:** 1 Department of Biological and Biomedical Sciences, North Carolina Central University, Durham, NC, United States; 2 Department of Psychiatry and Behavioral Sciences, Duke University Medical Center, Durham, NC, United States; 3 Howard Hughes Medical Institute, Chevy Chase, MD, United States; 4 Department of Neurobiology, Duke University Medical Center, Durham, NC, United States; 5 Biological and Biomedical Research Institute, North Carolina Central University, Durham, NC, United States

**Keywords:** alcohol use disorder, astrocyte, connexins (Cx), mechanisms of disease, neuroinflammation, therapeutic potential

## Abstract

Astrocytes are critical regulators of brain homeostasis and are known to be disrupted during alcohol use disorder (AUD). Here, we describe what is known about astrocytic dysfunction in AUD and the emerging literature supporting a mechanistic link to connexins in the pathophysiology of this disorder. We integrate what is known in the current literature and highlight areas where a lack of evidence leads to inconclusive interpretations. Overall, this is a comprehensive review of astrocytes and connexins in AUD, providing ideas and insights to basic and clinical audiences interested in pursuing future therapeutic mechanisms in AUD.

## Introduction: inflamed astrocytes are a hallmark of alcohol use disorder

Astrocytes mediate important events in neurotransmission and are vital regulators of the excitatory and inhibitory balance within the brain (1–5). This balance is disrupted by chronic alcohol exposure, contributing to the pathology of alcohol use disorder (AUD) and depression, the most common co-occurring disorder with AUD (6). Chronic ethanol exposure and withdrawal are also mechanistically linked to excitotoxicity in pyramidal neurons through the upregulation of glutamate receptors (7, 8). Excitotoxicity is known to exacerbate neuronal cell death and damage the neurovascular unit and blood-brain barrier (BBB) (9, 10). Neurovascular coupling and hemodynamic flow are also impacted by dysregulated water and ion homeostasis within the brain during AUD. These are all hallmarks of AUD, which are governed, at least in part, by astrocytes.

A multitude of studies have consistently linked inflammation and immune dysfunction to AUD. Cerebrospinal fluid studies reveal increased levels of pro-inflammatory cytokines such as IL-6 and MCP-1 in individuals with AUD (11, 12), and postmortem brain analyses showed that patients with AUD had increased levels of key inflammatory mediators, including pNFkB p65, IL-8, and IL-1β (13, 14). Clinical evidence also supports significant changes in astrocytes during AUD (15, 16). However, there are conflicting findings, due in part to the extraordinary heterogeneity in astrocyte phenotypes (17–21). A study that integrated genome-wide association study (GWAS) data with single-nucleus transcriptomics identified astrocytes as key contributors to the genetic architecture of AUD, with distinct astrocyte subtypes exhibiting alterations in homeostatic, inflammatory, ECM-modulating, and synapse-related transcriptional profiles (22). Other analyses revealed that astrocytes across the human prefrontal cortex, hippocampus, and amygdala all showed increased inflammatory and interferon signaling and that astrocytes are the primary contributors to gene expression changes in the brain due to alcohol dependence (23–25).

Interestingly, while some studies show elevated astrocyte activation in the orbitofrontal cortex and the caudate nucleus in individuals with AUD (26, 27), others report lower astrocyte numbers in the hippocampus of patients with AUD (28). Of note, many postmortem studies from decades ago had a very low sample size (for instance, 28 had N = 5/group), and methods to label and count astrocytes have improved since increasing the level of specificity in identifying subsets of astrocytes and more nuanced phenotypic distinctions. Regardless, the apparent contradictions in results suggest a dynamic process in which astrocytes may become overactivated in some regions while being depleted or dysfunctional in others in time-, dose-, and comorbidity-dependent ways.

When patients living with AUD were assessed for biomarkers, plasma glial fibrillary acidic protein (GFAP), a marker of astrogliosis, and neurofilament light chain, a marker of neuronal injury, were both elevated during periods of abstinence (29). In this same study, a PET tracer binding to MAO-B, an enzyme predominantly expressed in inflamed astroglia, showed that MAO-B binding was *inversely* correlated to AUD severity, withdrawal, and anxiety. Similarly, PET imaging of the translocator protein (TSPO), a marker of both micro- and astroglial inflammation (30), also showed reduced levels in the AUD patient group compared to controls (31). This pattern of astrocyte dysfunction and downregulation of astrocytic markers after years of alcohol use is the reverse of what is often seen after hours to weeks of ethanol exposure in preclinical models. This is also distinct from what has been observed in other brain disorders we often describe as inflammatory scenarios (such as brain injury or neurodegeneration). A reduction in TSPO and MAO-B here suggests that long-term, chronic ethanol exposure in human patients may lead to a unique phenotype compared to acute or week-long paradigms, which are often used in preclinical studies. Understanding and controlling the dysregulation of alcohol on astrocytes has the potential to impact both the neurobiological and behavioral maladaptations associated with AUDs (32–35).

Collectively, these findings indicate that astrocytes in AUD undergo complex, regional, and disease-state-specific structural, molecular, and functional changes. This convergence of clinical imaging, genetic, and postmortem evidence supports the conclusion that astrocytes are central to neurobiological and immune mechanisms underlying alcohol-related brain pathology. Thus, pursuing therapeutic targets that would improve astrocytic inflammation and concomitant loss of homeostatic function are imperative. However, studies to identify biomarkers and therapeutic targets in astrocytes are still in their nascent stages. Here, we outline evidence supporting the notion that connexins are key regulators of astrocytic function and dysfunction during AUD. We postulate that investigating connexins in astrocytes is worthwhile in our search for therapeutic targets to alleviate neuroinflammation during AUD. We review what is known about astrocytes in AUD and where questions remain due to a lack of evidence. We implore the field to take a careful look at astrocytic connexin mechanisms and to design future studies – from preclinical to human – to deepen our understanding of these mechanisms. This is all towards the ultimate goal of developing biomarkers and disease-modifying therapeutics for patients suffering from AUD.

## Ethanol causes disruptions in astrocytes and neurotransmission

**Figure 1:**
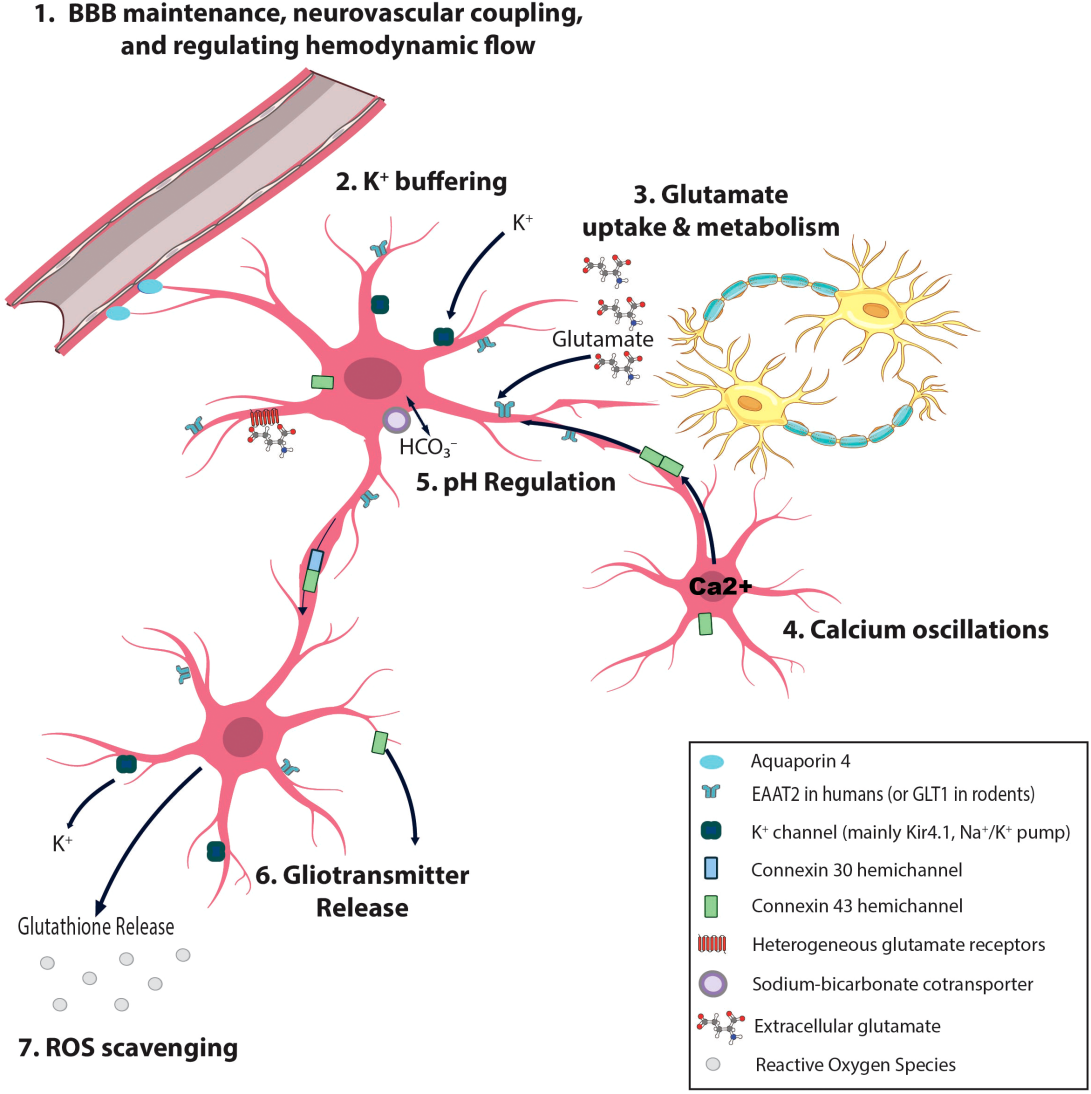
Homeostatic Astrocyte Functions. This schematic depicts some of the critical regulatory mechanisms of astrocytes in healthy brains which are known to be perturbed during AUD. They include: 1) BBB maintenance, neurovascular coupling, and regulating hemodynamic flow, 2) K+ buHering, 3) Glutamate uptake and metabolism, 4) Calcium oscillations, 5) pH regulation, 6) Gliotransmitter release, and 7) ROS scavenging. Key for all symbols is located in the bottom right..

Astrocytic processes closely interact with pre- and post-synaptic neurons to form the tripartite synapse, where they modulate neurotransmission by taking up extracellular neurotransmitters, releasing gliotransmitters, and having direct contact with neurons at dendritic spines (**Figure 1**) (2, 36–42). One example gliotransmitter is ATP, which is released through astrocytic connexon hemichannels into the extracellular space (more on hemichannels later), where it is converted to adenosine. Adenosine mediates significant aspects of the sedative/hypnotic effects of ethanol through the Adenosine_2A_ Receptor (A2AR) (43). This receptor is expressed predominantly on astrocytes, neurons, and endothelial cells in homeostatic conditions and upregulated in microglia during inflammation (44–46). Astrocytes also clear glutamate from the synaptic cleft to maintain excitatory homeostasis (47–50), where they take up glutamate primarily through the type 2 excitatory amino acid transporter (EAAT2), also known as glutamate transporter 1 (GLT-1) in rodents (1, 47, 50). In rodent models of alcohol dependence, chronic ethanol exposure decreases GLT-1 and the cysteine glutamate antiporter xCT expression, which impairs clearance of extracellular glutamate (32, 51–54). Elevated extracellular glutamate levels also follow chronic ethanol exposure in humans and rats in periods of withdrawal (7, 8, 55, 56). This was observed by magnetic resonance spectroscopy in humans, and the mechanism was confirmed to be driven by upregulated glutamate receptors in rodent hippocampal slices.

Normally, astrocytes prevent excitotoxicity by rapidly converting glutamate to glutamine through the action of glutamine synthetase (1, 47, 50, 57). This glutamine is subsequently released into presynaptic neurons, where it is converted into glutamate and repackaged into synaptic vesicles for future release (2). Much more about glutamate metabolism is elegantly reviewed by Adermark and Bowers in their review on astrocytes in AUD (1).

In the context of AUD, ethanol exposure decreases glutamine synthetase activity, which impairs glutamate/glutamine metabolism (58). In the prefrontal cortex and hippocampus of male Sprague Dawley rats, the downregulation of GLT-1 and concomitant increase in extracellular glutamate leads to NFkB-dependent neuroinflammation (59), which is further exacerbated by increased ROS production (60). Ethanol exposure also induces astrocytic swelling, which has been linked to impaired hemodynamic flow and increased cell death within the CNS (57, 61). Astrocytic swelling induces these glia to release taurine, a potent regulator of dopamine and the rewarding effects of ethanol within the mammalian brain (62–64). These are key discoveries in how astrocytes mediate dysfunction within AUD and ethanol exposure models (**Table 1**).

**Table 1:** Summary of key findings from current literature and the model and organism used.

Category	Key Finding	Model	Organism	Citation
Critical functions of astrocytes which inform the ethanol-related findings	Astrocytic glypican-4 and -6 induce formation of functional excitatory synapse by promoting surface clustering of GluA1 AMPA receptors	in vivo glypican-4 KO mouse, in vitro rat RGC culture, ex vivo hippocampal slice	Mouse, rat	Allen 2012 (36)
	Immature astrocytes secrete TSP-1 and -2 which promote fomrmation of ultrastructurally synapses that are presynaptically active but postsynaptically silent during development	in vivo TSP-1/TSP-2 deficiency mouse, in vitro rat RGC culture, in vitro cortical astrocyte culture	Mouse, rat	Christopherson 2005 (37)
	Neuronal α2δ-1 mediates TSP-dependent excitatory synaptogenesis	in vivo TSP1/2 double KO mice, in vivo α2δ-1 overexpress mouse, in vitro rat RGC neuron culture, in vitro HEK293 cells	Mouse, rat, human	Eroglu 2009 (38)
	Astrocytic process contact stabilizes dendritic protusions and promotes spine maturation via Rac1-dependent astrocytic motility and ephin/Eph-dependent neuron-astrocyte signaling	ex vivo mouse hippocampal slice	Mouse	Nishida and Okabe 2007 (39)
	Astrocytic hevin promotes excitatory synaptogenesis and synapse maturation, and astrocytic SPARC antagonizes hevin-mediated synapse formation.	in vitro rat RGC culture, in vivo hevin KO and SPARC KO mice	Mouse, rat	Kucukdereli 2011 (41)
	Astrocytic adhesion activates neuronal integrins, which triggers PKC signaling that promotes excitatory synaptogensis	in vitro rat hippocampal neuron with astrocyte contact	Rat	Hama 2004 (42)
	Acute heavy ethanol-induced NREM sleep requires adenosine A2A receptor signaling in the brain	in vivo ethanol-induced sleep in A2AR KO mice	Mouse	Fang 2017 (43)
	Cell-type expression atlases: https://brainrnaseq.org/?3165123080=3121471639. Used to describe the cell types when *Adora2a* or *ADORA2A* is expressed in this review.	in vitro purification of mouse cortical CNS cell types	Mouse	Zhang 2014 (44)
		ex vivo purification of human CNS cell types, in vitro human astrocyte and rat neurons co-culture	Human, rat, mouse	Zhang 2016 (45)
		in vivo mouse microglia, ex vivo human brain tissue	Mouse, human	Bennett 2016 (65)
	Aging induced A1-like reactive astrocyte phenotype driven by microglial cytokines with region-specific transcriptional changes	in vivo mouse, ex vivo brain tissues	Mouse	Clarke 2018 (20)
	Classical-activated microglia induced A1-like astrocytes that led to neuronal and oligodendrocyte death via astrocytic secretion of Il-1a, TNFa and C1q	in vitro mouse astrocyte culture, in vivo mouse and rat, ex vivo human postmortem brain tissue	Mouse, rat, human	Liddelow 2017 (21)
Ethanol impact on astrocytes known mechanisms	5-week voluntary ethanol exposure in alcohol-preferring rats reduced GLT-1 and xCT in the amygdala and hippocampus.	in vivo rat chronic ethanol exposure	Rat	Aal-Aadoba 2015 (51)
	6-week voluntary ethanol exposure in alcohol-preferring rats reduced GLT-1 expression and increased BDNF, Arc, and p-nNOS signaling in the nucleus accumbens shell.	in vivo rat chronic ethanol exposure	Rat	Alhadad 2020 (52)
	7-day ethanol exposure followed by methamphetamine administration downregulated GLT-1 in the striatum and hippocampus, with greater reductions after combined exposure.	in vivo rat ethanol and methamphetamine exposure	Rat	Alsheri 2017 (53)
	5-week ethanol exposure in alcohol-preferring rats increased extracellular glutamate and reduced GLT-1 in the nucleus accumbens	in vivo rat chronic ethanol exposure	Rat	Das 2015 (32)
	Acute alcohol withdrawal increased prefrontal glutamate levels alcohol-dependent humans and rats and glutamate:glutamine ratio in rat, which would normalize after several weeks of abstinence	in vivo human and rat alcohol withdrawal	Human, rat	Hermann 2012 (55)
	4-day heavy ethanol exposure increased Ca2+/calmodulin-dependent kinase activity and Na+-dependent glutamate uptake in cortical astrocytes	in vitro rat cortical astrocyte culture	Rat	Smith and Navratilova 1999 (56)
	7-day heavy ethanol exposure followed by withdrawal increased glutamatergic synaptic activity and induced excitotixic CA1 pyramidal neuron injury via AMPA and mGluR5 signaling	ex vivo rat organotypic hippocampal slice culture	Rat	Gerace 2019 (7)
	7-day heavy ethanol exposure followed by withdrawal increased GluA1 expression and AMPAR-mediated excitability in CA1 neurons, which contribute to excitotoxic cell death	ex vivo rat organotypic hippocampal slice culture	Rat	Gerace 2021 (8)
	High ethanol exposure reduced glutamine synthetase activity and protein synthesis, which led to impairment of glutamate:glutamine metabolism in glial cells.	in vitro embryonic chick cerebral hemisphere glial enriched culture	Chick	Davies and Vernadakis 1984 (58)
	4-week moderate ethanol exposure induced astrocytic activation, NF-kB signaling and reduced GLT-1 expression that increases neuroinflammation	in vivo rat ethanol exposure	Rat	Villavicencio-Tejo 2021 (59)
	4-week taurine treatment bidirectionally altered striatal DAT expression and dopamine uptake. High-dose taurine reduced DAT and improved working memory performance	in vivo rat with taurine treatment	Rat	Chen 2018 (62)
	Hypotonic swelling in neonatal rat astrocyte cultures triggers anion channel-mediated release of glutamate and taurine, independent of Na+-dependent uptake system	in vitro astrocyte cultures	Rat	Kimelberg 1990 (63)
	Acute ethanol exposure induces astrocyte swelling and taurine release that activates glycine receptors to elevate dopamine in the nucleus accumbens	in vitro ethanol exposure in astrocyte culture, in vivo rat nucleus accumbens microdialysis	Rat	Adermark 2011 (64)
	Acute and chronic ethanol exposure increases astrocyte swelling and enhances volume expansion during hyponatremic conditions	in vitro ethanol exposure in rat cortical astrocyte culture	Rat	Aschner 2001 (66)
	Glutamate induces astrocyte swelling via mGluR5 activagtion and AQP4 upregulation that contribute to ischemia-associated brain edema	in vitro glutamate exposure in neonatal rat cortical astrocyte culture, in vivo tMCAO rat	Rat	Shi 2017 (67)
	Cerebral ischemia induces swelling at astrocytic processes that compresses capillaries and contributes impaired reperfusion and brain injury	in vivo rat global cerebral ischemia model	Rat	Dietrich 1986 (61)
	Multiple regression revealed SNPs in alcohol metabolism genes are associated with alcohol consumption and dependence, with ADH1B rs1229984 showing the strongest effect.	Epidemiological study assessing SNPs and alcohol dependence behavior via self-report survey	Human	Sherva 2009 (68) (comparative study)
	Astrocytic ALDH1A1 and SIRT2 mediate MAO-B-dependent putrescine metabolism to produce tonic GABA in astrocytes, a pathway implicated in memory impairment.	in vitro astrocyte culture	Mouse	Bhalla 2023 (69)
	Acute low-dose ethanol exposure activated astrocytic ALDH2 to produce acetate, which increased cerebellar GABA and tonic inhibition, causing impairment in balance and coordination skills	in vivo mouse ethanol exposure with astrocyte-specific ALDH2 KO, ex vivo cerebellar slice, in vitro astrocyte culture	Mouse	Jin 2021 (70)
	High ethanol exposure reduced glutamine synthetase activity and protein synthesis, which led to impairment of glutamate:glutamine metabolism in glial cells.	in vitro embryonic chick cerebral hemisphere glial enriched culture	Chick	Davies and Vernadakis 1984 (58)
	1-5 day ethanol exposure dose-dependently inhibited proliferations of human astrocytes	in vitro ethanol exposure in adult human astrocyte cultures	Human	Kane 1996 (71)
	Acute moderate ethanol exposure activated HSF1 and induced heat-shock and metabolic gene expression in astrocytes via heat-shock pathway	in vitro ethanol exposure in cortical astrocyte culture	Mouse	Pignataro 2013 (72)
	AUD is associated with widespread cell type-specific transcriptomic changes across the cortex, with strongest dysregulation seen in the microlia and astrocyte subtypes that are linked to genetic risks of AUD	ex vivo postmortem human cortex	Human	Warden 2026 (22)
	Alcohol dependence resulted in cell type specific transcriptomic changes, with greatest differential expression in astrocytes, oligodendrocytes and microglia, particular in neuroinflammatory-related genes	ex vivo postmortem human prefrontal cortex	Human	Brenner 2020 (25)
	A new transcriptome profiling approach revealed alcohol dependence changed gene expression in the brain though epigenetic mechanisms, including reduced DNA methylation and increased H3K4 trimethylation.	ex vivo postmortem human brain tissue	Human	Ponomarev 2012 (24)
	Alcohol abuse were found to link to 37% reduction in hippocampal glial cells, including astrocytes, oligodendrocytes and microglial cells, with no neuronal loss	ex vivo postmortem human hippocampus	Human	Korbo 1999 (28)
	PET imaging of astrocytic MAO-B showed abstinence in AUD participants resulted in variable MAO-B binding, which is inversely correlated with AUD severity, and increased plasma GFAP and NF-L	Clinical study using PET and MRI to assess MAO-B binding and self-reported symptoms in AUD patients, confounds also due to cigarette smoking	Human	Best 2025 (29)
	Meta-analysis from 3 PET studies showed no significant heterogeneity. Three studies all showed that TSPO binding was lower in alcohol group than in control group.	Meta-analysis of biomakers in CSF, PET and postmortem studies on AUD	Human	Adams 2023 (30)
	Alcohol withdrawal in ADP showed reduced TSPO in hippocampus, which was positively correlated with poorer verbal memory performance	Clinical study using PET to assess microglial TSPO expression and cognitive performance in alcohol dependence	Human	Kalk 2017 (31)
	Chronic heavy alcohol consumption was characterized by 639 differential expressed genes that are linked to increased inflammation, hypoxia and stress pathways, and decreased neurogenesis and myelination, with many related to astrocytes	ex vivo postmortem human hippocampus	Human	McClintick 2013 (23)
Connexis mediate important changes in astrocytes after chronic ethanol exposure	Clinically-relevant ethanol concentrations opens Cx43 and Panx1 in astrocytes, which promotes ATP and glutamate release via inflammatory signaling pathways	in vitro mouse cortical astrocyte culture	Mouse	Gomez, Alvear 2024 (73)
	2-week heavy ethanol exposure in adolescence opens Cx43 and Panx1 in a MAPK-dependent manner to influence neuroinflammation	in vivo rat ethanol exposure, ex vivo hippocampal slice	Rat	Gomez 2018 (74)
	2-week heavy ethanol exposure in adolescence opens Cx43 and Panx1 in a MAPK-dependent manner to influence neuroinflammation	in vivo rat ethanol exposure, ex vivo hippocampal slice	Rat	Gomez 2018 (74)
	Prenatal moderate-heavy ethanol exposre increased astrocytic Cx30 and Cx43 expression in the hippocampus, which contributes to brain hyperexcitability and seizure susceptibility.	in vivo mouse ethanol exposure, ex vivo hippocampal and cortical slices	Mouse	Ramani 2016 (75)
	Clinically-relevant ethanol concentrations opens Cx43 and Panx1 in astrocytes, which promotes ATP and glutamate release via inflammatory signaling pathways	in vitro mouse cortical astrocyte culture	Mouse	Gomez, Alvear 2024 (73)
	Acute ethanol exposure triggered Ca2+-dependent activation of astrocytic Cx43 and Panx1 hemichannels, leading to sustained intracellular Ca2+ elevation and increased ATP and glutamate release	in vivo cortical astrocyte culture of mouse undergone ethanol exposure, ex vivo mouse brain slices, in vitro HeLa cells	Mouse	Gomez, Garcia-Rodriguez 2024 (76)
	7-week moderate ethanol exposure in ALDH2-2 mouse reduced total Cx43 and promoted ventricular arrhythmias with action potential prolongation, which was consistent with QT prolongation and increased arrhythmia risk in ALDH2-deficient humans	in vivo ALDH2-2 knock-in mouse undergone ethanol exposure, in vivo human, ex vivo mouse heart	Human, mouse	Lee 2023 (77)
Astrocytic connexins in other related psychiatric disorders	9-day repeated morphine exposure induced naloxone-precipitated withdrawal behaviors, which were reduced by hippocampal astrocytic Cx43 inhibition.	in vivo rat morphine exposure	Rat	Darvishmolla 2022 (78)
	Astrocytic connexin 43 knockout displays anhedonia, despair, and anxiety-like phenotypes whereas Cx43 overexpression rescues ATP deficit and depression-like phenotypes in CSDS mice	in vivo mouse	mouse	Wang 2025 (79)
	Chronic social defeat stress decreased astrocytic Cx30 and Cx43 in the mPFC and hippocampus, which reduced neuronal activity and promoted depressive-like behaviors	in vivo chronic social defeat stress mouse, ex vivo brain slices	Mouse	Huang 2019 (80)
	Gene expression analysis showed reduction in astrocytic connexins Cx30 and Cx43 in the dorsolateral prefrontal cortex is associated with suicide, and identified Sox9 as a regulator of Cx30 expression	ex vivo human postmortem dorsolateral prefontal cortex, in vitro rat astrocyte culture+human HEK-293 cells	Human, rat	Ernst 2011 (81)
	H3K9me3-mediated epigenetic repression of astrocytic Cx30 and Cx43 is associated with widespread gap-junction dysfunction across many brain regions in depressed suicide subjects	ex vivo human postmortem cortical and subcortical tissues	Human	Nagy 2017 (82)
Additional Human evidence of inflammation in AUD	28-day of alcohol withdrawal showed elevated CSF MCP-1 level in alcoholics compared to healthy individuals, which was linked to alcohol-induced liver inflammation	Clinical cross-sectional study assessing CSF MCP-1, I1-B, TNFa in alcoholics	Human	Umhau 2014 (12)
	Increased neuroimmune death receptor signaling in the hippocampus of AUD patients and adolescent binge-drinking rats correlated with coordinated activation of caspase-mediated cell death pathways and increased pNF-kB and IL-8	ex vivo postmortem AUD human hippocampal tissues, in vivo rat ehtanol exposure	Human, rat	Liu 2021 (13)
	Ethanol exposure induced IL-1B–NALP1/3 inflammasome signaling that mediates ethanol-impaired neurogenesis in neurons and astrocytes	ex vivo rat organotypic hippocampal-entorhinal cortex brain slice cultures, ex vivo postmortem human brain tissue	Human, rat	Zou and Crews 2012 (14)
	White vs grey matter astrocyte comparison showed distinct molecular and functional differences	in vivo mouse, in vivo postmortem human with ex vivo brain slice	Mouse, human	Bocchi 2025 (17)

These findings have direct mechanistic links to connexins. In cell culture and rodent models, chronic ethanol exposure significantly increases molecular flux through connexon 43 (Cx43) hemichannels to induce toxic levels of extracellular glutamate and ATP. This drives astrocyte-mediated inflammation and tissue damage (73, 74, 76). In other disease models (not AUD), astrocytic swelling can be reduced by blocking Cx43 channel opening (61, 66, 67, 83), which promoted improved CNS blood flow and neuronal health. This has not yet been tested in AUD, but would be an exciting area of inquiry. There is also a remarkable body of emerging evidence that astrocytes can transfer mitochondria to neurons. Studies in the CNS and other organ systems have shown this is mediated by Cx43-dependent tunneling nanotubes and connexosome formation (84–86). Through a wide variety of mechanisms, Cx43 is a key player in mediating inflammation, metabolic support of neurons, BBB health, and hyperexcitability during homeostasis and a variety of pathologies. However, this is not the only connexin astrocytes express, and there is still so much to learn about connexin-mediated effects in astrocytes during AUD. To further dive into these fascinating connections, we will now review connexin biology and what is already known about how connexins influence astrocytes (87).

## Important background to understand connexin-mediated cellular communication

The human genome encodes 21 distinct connexin isoforms, named according to their molecular weight in kiloDaltons. Six of these connexins assembled into a hexameric ring are called a connexon. When trafficked to the cell membrane, connexons form a selectively permeable hemichannel for influx and/or efflux. Hemichannels can be formed homomerically (made of the same six connexins) or heteromerically (made of different connexins within the connexon). These hemichannels can function independently as hexamers at membranes to facilitate the exchange of ions, metabolites, and other diffusible molecules between the cytoplasm and the extracellular space. They can also dock with another connexon from an adjacent cell, forming a gap junction channel to facilitate intercellular communication (88–92). These gap junctions can form homotypically (same connexon types on both sides) or heterotypically (different connexon types on either side). These gap junctions can dock between cells of one identity (e.g. astrocyte to astrocyte) or facilitate cellular crosstalk between distinct cell identities (e.g. astrocyte to oligodendrocyte) (88, 93). They are regulated by their expression levels, trafficking patterns to membranes, and can also display open or closed conformations dependent on a myriad of factors (pH, calcium concentrations, phosphorylation status, voltage changes, and beyond) (89, 91, 94).

Changes in connexin expression during development are observed across various cell types, with specific connexin isoforms being dynamically regulated to support diverse functions (95–97). There is robust evidence that connexin expression and activity vary according to cell identity, developmental stage, and disease state (93, 96–101) - even within a single tissue - indicating precise regulatory mechanisms that operate via multiple mechanisms (74, 76, 98, 99, 102, 103).

## Astrocytic connexins 43 and 30 occupy discrete and overlapping niches

Astrocytes are heterogeneously distributed across the brain cortex and subpial spaces. There are unique molecular and behavioral phenotypes across diverse subpopulations depending on their origins, developmental timing, which region they occupy, if they are within grey matter vs. white matter, if they are within the perivascular niche or not, and if they are in brains which have experienced injury or disease (20, 57, 102, 104). Several studies using immunohistochemical, immunocytochemical, freeze-fracture electron microscopy, *in situ* hybridization, and single-cell RNA Sequencing techniques have demonstrated significant expression of three connexins in astrocytes: Cx43, Cx30, and Cx26 (101, 105–108). Cx43 is expressed in astrocytes throughout the rodent brain during early development and Cx43 expression levels remain the highest expressed connexin throughout adulthood. Cx43 proteins are found ubiquitously in astrocytes both as hexamers in astrocytic processes near neuronal somas, dendrites, and synapses, as well as astrocyte-astrocyte gap junctions (108, 108). Conversely, Cx30 is not expressed until mammals develop postnatally and is primarily localized to perivascular astrocytic endfeet, with no expression in white matter astrocytes (101, 109). The localization of Cx30 near blood vessels and exclusive postnatal expression imply that Cx30 may play astrocytic subtype-specific roles in perivascular astrocytes to support the postnatal BBB. Both Cx43 and Cx30 can be found in homomeric/homotypic gap junctions or bound to one another. Interestingly, Cx26 expression in astrocytes begins postnatally and remains throughout adulthood, where it is predominantly found in heterotypic gap junctions across cell types, binding astrocytes with oligodendrocytes, neurons, and leptomeningeal cells. Thus, Cx26 is thought to contribute to cellular cross-talk within the brain (100, 101, 105, 109–112). There are studies linking Cx26 to auditory impairments, breast cancer, and other disorders outside of the CNS; however, we found no connection thus far of Cx26 to AUD. Given this fact and the lack of specificity to astrocytes, we have focused less on Cx26 in the subsequent portions of this review. However, we do not exclude the possibility this connexin could have important roles in AUD, simply that it has not yet been reported.

## Astrocyte connexins are critical regulators for ionic homeostasis

Regulating ionic flux is pivotal to astrocytes and their impact on the brain. Astrocytic calcium transient waves are primarily initiated by the activation of Gαq-coupled receptors, which trigger the breakdown of phosphatidylinositol 4,5-bisphosphate (PIP_2_) into inositol 1,4,5-trisphosphate (IP_3_), causing Ca^2+^ release from intracellular stores into the cytoplasm (1, 113, 114). This Ca^2+^ signaling can propagate to neighboring astrocytes through Cx30 and Cx43 gap junctions to regulate the rate of astrocytic gliotransmitter release and uptake (1, 99, 115, 116). Gap junctions are the primary mechanism of intracellular Ca^2+^ waves and also contribute to ATP release, thereby amplifying the extracellular purinergic mechanism of Ca^2+^ waves (92, 99, 114, 116). Astrocytes also regulate extracellular pH, K+, and glutamate levels through direct intercellular communication (40, 93, 117, 118). Extracellular K+ is essential in determining the resting membrane potential of neurons and astrocytes, and the efficient clearance of K+ from the extracellular space is critical for maintaining brain homeostasis and preventing neuronal hyperexcitability (119). Glutamate/glutamine metabolism and signaling are also key to protecting against hyperexcitability, as described before.

Astrocytes also form an extensive gap junction-coupled syncytium that connects cells into an electrically isopotential network spanning the CNS, functioning as a brain-wide “astro-grid” that supports coordinated ionic and metabolic homeostasis (120). This syncytium can also be formed between astrocytes and other glial cell types, such as oligodendrocytes and ependyma cells (120). The astrocytic syncytium, formed through gap junction coupling, can function like one electrophysiologically unified system. This connectivity through connexins enables the high efficacy uptake and redistribution of excess extracellular K+ across a robust astrocytic network (111, 121), and proper distribution of many other ions, metabolites, and signaling molecules including cAMP, IP_3_, Ca^2+^, glucose, and glutamate (122, 123). In instances where there is uncoupling of astrocytes, there are disruptions in synaptic transmission and plasticity (123). Taken together, this is robust evidence implicating a crucial role of connexins in mediating cell-to-cell communication in astrocytes, which has a profound influence on brain health.

## Astrocytic connexins drive critical phenotypes in AUD

**Figure 2:**
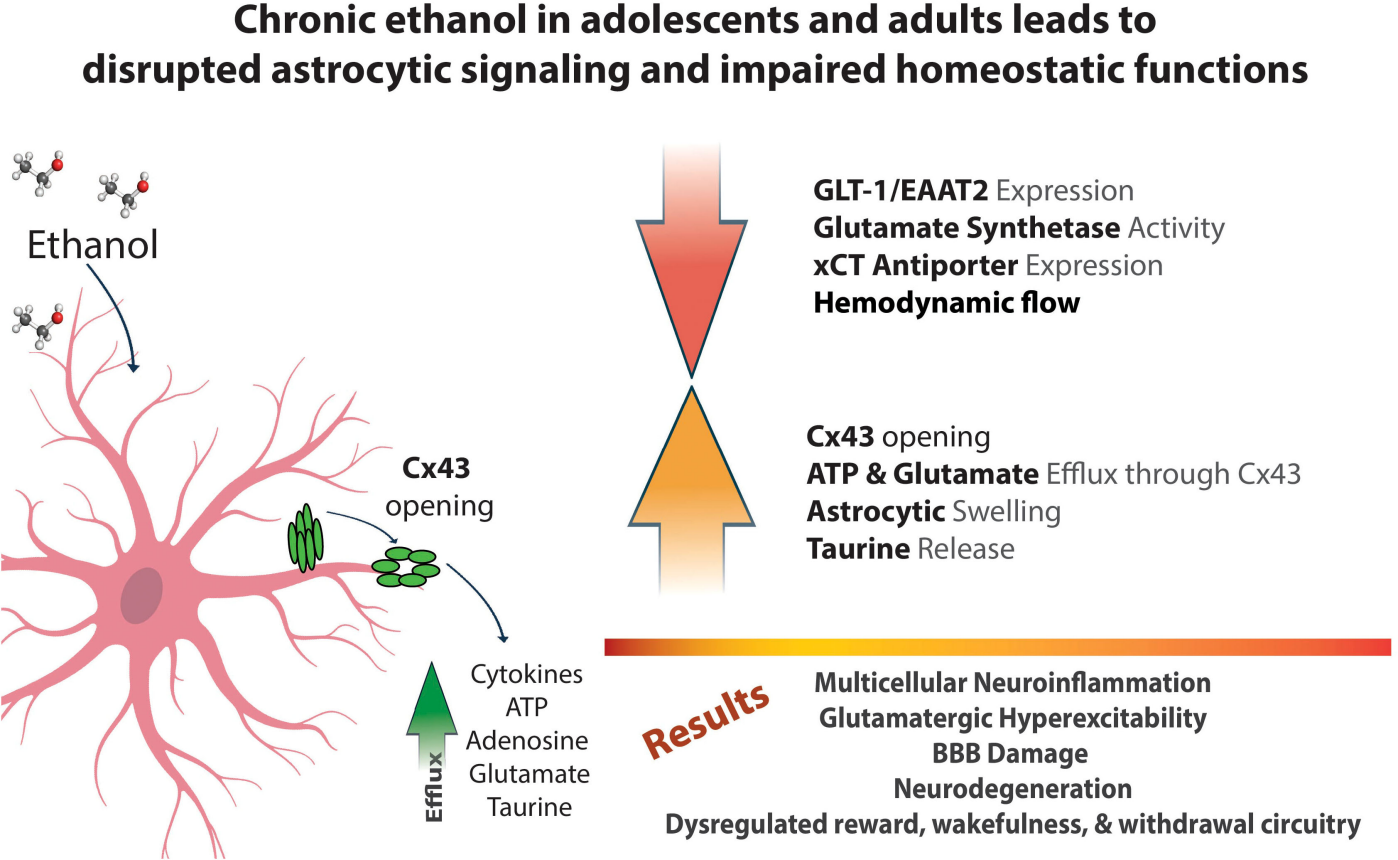
Chronic ethanol exposure in adolescents and adults leads to disrupted astrocytic signaling and impaired homeostatic functions. Red arrow down represents the downregulation, decreased, or lowered functions during AUD and the orange arrow up indicates the increased, upregulation, or more prevalently observed functions during AUD. The green arrow up indicates the molecules which have been shown to have increased efflux through Cx43 during AUD. The bottom right shows the pathological implications of these mechanisms and is labeled by “results”.

Connexins are emerging as critically important proteins that regulate astrocytic behavior and the pathogenesis of AUD (**Table 1**). Most research on astrocytic connexins and AUD has concentrated on the impact of ethanol on Cx43 expression and activity. This has revealed a role for Cx43 in alcohol-induced neurotoxicity, neuroinflammation, and disrupted astrocytic functions (**Figure 2**) (73–76). In rodent and cell culture models, alcohol exposure increased Cx43 hemichannel opening, which led to increased efflux of ATP and glutamate, and therefore excitotoxicity (73, 74, 76). Furthermore, a recent transcriptomic study of the prefrontal cortex of adult mice following chronic alcohol consumption focused on prominent alterations in extracellular matrix and Ca^2+^-related genes, but a deeper dive into the differentially expressed genes excel files showed significant enrichment for transcripts encoding Cx43, Cx30, and other connexin proteins as well (124).

A different murine model of infant prenatal ethanol exposure displayed increased connexin protein levels in the cortex (Cx30) and hippocampus (Cx30 and Cx43) neurons (75). This resulted in an increased prevalence of sharp wave activity in the hippocampus, which was reduced to normal after application of two broad-spectrum gap junction blockers. This discrepancy between models, where some observe only activity changes and some observe enrichment of protein levels at the membranes, underscores the complexity of connexin regulation across different cell types, regions, and developmental states. Ethanol exposure mediates unique impacts on astrocytic connexins depending on whether the exposure is before, during, or after the critical time periods during which Cx30 and Cx43 occur developmentally. In established, mature astrocytes, ethanol regulates the opening or closing the channels. Conversely, in immature astrocytes, connexin expression levels may be more prone to being influenced by the milieu in which they are developing. Ethanol’s impact on connexin activity vs. abundance is also dependent on brain region. Furthermore, these studies suggest connexins regulate circuit excitability, and other studies imply Ca2+ dysregulation in astrocytes to be prominent, but definitive evidence for which ions, metabolites, and messengers are involved in each context would be valuable information for future therapeutic targeting strategies to leverage these mechanisms.

In a study conducted on human postmortem tissue, investigators observed that when compared to control individuals, there was a significant decrease in Cx43 protein expression in the orbitofrontal cortex in patients who met clinical criteria for Major Depressive Disorder (MDD), AUD, or both (125). However, they did not label or isolate astrocytes, so it is unclear if this downregulation is due to decreased Cx43 within astrocytes fewer astrocytes overall. Furthermore, this study measured absolute abundance but did not evaluate membrane localization or activity levels of Cx43. Given the myriad of levels by which connexins are regulated, future studies could improve interpretability by including assays to measure abundance, localization, *and* activity levels within each brain region and ethanol exposure paradigm.

There are no current findings linking single nucleotide polymorphisms (SNPs) in Cx30 or Cx43 directly to AUD. However, there are two genome-wide association studies (GWAS) that indicate SNPs in aldehyde dehydrogenase, a superfamily of detoxification enzymes, are associated with AUD (68, 77). This is particularly fascinating given that ALDH1A1, the protein encoded by one of the implicated genes, is a ubiquitously used cell marker for astrocytes. ALDH1A1 is known to have key functions in synthesizing GABA and regulating excitatory/inhibitory tone in the brain (69). Furthermore, when a mouse line developed to express the known ALDH2 SNP was subjected to chronic, moderate alcohol use, ALDH2 mutants displayed a downregulation of Cx43 in the heart, which contributed to ventricular arrhythmias (68). Astrocytic ALDH2 has already been implicated in driving balance and coordination issues associated with ethanol intoxication, as driven by aberrant GABA levels in the cerebellum (70). Taken together, although preliminary, these findings are important links between the human genomic and epidemiology discoveries with mechanistic murine studies, which mutually support each other in implicating ethanol metabolism, aldehyde dehydrogenase, and connexins in altering the electrophysiological properties of the heart and brain. How this mechanism may be at play in the brain during AUD remains unknown and would be an interesting area of inquiry for future studies.

## Connexins in astrocytes mediate critical phenotypes during psychiatric disorders

Other intriguing pieces of the puzzle involve the role of hippocampal astrocytic Cx43 in morphine dependence and depression-like phenotypes. While a different substance, many studies have connected overlapping circuitry mediating chemical dependence and withdrawal across different substances. This particular study demonstrated that inhibiting astrocytic Cx43 in morphine-treated rats reduced withdrawal symptoms, suggesting a role for astrocytic connexin-mediated gliotransmitter release in addictive behaviors (78). An additional study in rodent models observed increased despair and anhedonia phenotypes in Cx43 knockout mice, and that Cx43 overexpression in the medial prefrontal cortex rescued ATP levels and depression-like behaviors in mice susceptible to chronic social defeat stress (79). While not in the context of ethanol, these studies give support to the notion that astrocytic connexins are critical to glia/neurotransmission and downstream emotion-related behaviors (87).

Are connexins and their signaling cascades involved in these mechanisms? Do connexins alter downstream behaviors like dependence and withdrawal during AUD as they do in morphine? Given that glutamate receptor-mediated excitotoxicity in the hippocampus is a critical mechanism in withdrawal and astrocytes alter glutamate and taurine-mediated dopamine signaling in key areas connected to withdrawal, one could logically surmise there is potential for a mechanistic connection here. It would be of great interest to conduct connexin knockout and overexpression studies in AUD models.

Of note, while there is a plethora of emerging evidence implicating Cx43 in AUD and other disorders of addiction and withdrawal, there is a notable gap in our understanding of the specific contribution(s) of Cx30 in AUD. While Cx30 is known to be co-expressed with Cx43 in astrocytes and to play key roles in glial communication, glutamate clearance, and cell permeability, very little is known about how this protein is influenced by or mediates the effects of AUD (101, 102, 106, 109, 126). There are a few studies that connect Cx30 to suicide completion, chronic insomnia disorder, and depressive-like behaviors in humans and rodents, however, there is much more to elucidate when it comes to the mechanisms by which Cx30 exerts its roles in AUD (80–82). In particular, the role of Cx30 in perivascular astrocytes and how this may or may not impact BBB phenotypes during AUD would be of particular interest given its expression patterns. Interestingly, a transgenic inducible cre-mouse line to target the Cx30 (*Gjb6)* promoter already exists and could be of great utility in exploring this subpopulation compared to all astrocytes (127).

## Synthetic electrical synapses are exciting emerging tools we could leverage in our studies

Using synthetic and ectopic electrical synapses to modulate neuronal activity through ‘synaptic engineering’ is a thrilling area of emerging research. This has been reviewed by three leading pioneers in this superb 2024 review (128). While the use of ectopic Cx36 or other genetically engineered connexins is efficacious in altering *C. elegans* and murine neuronal electrophysiology and behavior, this work has yet to extend to targeting astrocytes (129, 130). Theoretically, the same tools could be applied to increase expression of connexon hexamers and gap junctions in astrocytes, specifically, or in concert with other cell types. Similarly, if overexpression or increased activity were implicated in a particular disorder, dominant-negative approaches to close these pores could be leveraged. This provides an exciting parallel endeavor within an adjacent field that could come into play down the road.

## Conclusions and future directions

Future research should investigate the age-, region-, paradigm-, and species-specific effects of ethanol on Cx43 and Cx30 abundance and activity in astrocytes. By further examining the processes by which ethanol influences Cx30 and Cx43 activity, researchers may uncover parallel or distinct pathways that exacerbate alcohol withdrawal symptoms, promote dependence, alter mitochondrial transport from astrocytes to neurons, and/or mediate inflammatory effects within the brain.

There is also exciting new work in connexin biology to engineer synthetic electrical synapses, which could prove invaluable in understanding the mechanisms of neuroinflammation and neurotoxicity in AUD and novel therapeutic targets to improve patient outcomes. While the current work in the field appears to focus on neuron-neuron connections, expanding these studies to include modulating astrocytic activity is an exciting future frontier to explore.

A preponderance of evidence suggests that connexins in astrocytes are vitally important to brain health. Furthermore, many emerging studies link Cx43 to key cellular mechanisms of pathogenesis in AUD and other addiction-related psychiatric disorders. However, future studies are still required. We implore the field to use a robust integration of approaches to test connexin abundance, localization, activity, and downstream effects in AUD and to include Cx30 in their investigations. These future findings could expand our understanding of the molecular underpinnings of AUD and potentially identify new therapeutic targets to disrupt the maladaptive cellular cascades that drive adverse outcomes in AUD.
